# Visualization of clinically silent, odontogenic maxillary sinus mucositis originating from periapical inflammation using MRI: a feasibility study

**DOI:** 10.1007/s00784-023-04986-4

**Published:** 2023-04-11

**Authors:** Egon Burian, Georg Feuerriegel, Nico Sollmann, Gintare Burian, Benjamin Palla, Magdalena Griesbauer, Caspar Bumm, Monika Probst, Meinrad Beer, Matthias Folwaczny

**Affiliations:** 1 Department of Diagnostic and Interventional Neuroradiology, Klinikum Rechts Der Isar, School of Medicine, Technical University of Munich, Munich, Germany; 2 Department of Diagnostic and Interventional Radiology, University Hospital Ulm, Ulm, Germany; 3 Department of Diagnostic and Interventional Radiology, Klinikum Rechts Der Isar, School of Medicine, Technical University of Munich, Ismaninger Str. 22, 81675 Munich, Germany; 4 TUM-Neuroimaging Center, Klinikum Rechts Der Isar, Technical University of Munich, Munich, Germany; 5 Department of Prosthodontics, LMU University Hospital, Ludwig-Maximilians-University, Munich, Germany; 6 Department of Oral and Maxillofacial Surgery, University of Illinois, Chicago, IL USA; 7 Department of Restorative Dentistry and Periodontology, LMU University Hospital, Munich, Germany

**Keywords:** Magnetic resonance imaging, Periapical inflammation, Periapical osteolysis, Mucositis

## Abstract

**Objectives:**

Maxillary sinus mucositis is frequently associated with odontogenic foci. Periapical inflammation of maxillary molars and premolars cannot be visualized directly using radiation-based imaging. The purpose of this study was to answer the following clinical question: among patients with periapical inflammatory processes in the maxilla, does the use of magnetic resonance imaging (MRI), as compared to conventional periapical (AP) and panoramic radiography (OPT), improve diagnostic accuracy?

**Methods:**

Forty-two subjects with generalized periodontitis were scanned on a 3 T MRI. Sixteen asymptomatic subjects with mucosal swelling of the maxillary sinus were enrolled in the study. Periapical edema was assessed using short tau inversion recovery (STIR) sequence. Apical osteolysis and mucosal swelling were assessed by MRI, AP, and OPT imaging using the periapical index score (PAI). Comparisons between groups were performed with chi-squared tests with Yates’ correction. Significance was set at *p* < 0.05.

**Results:**

Periapical lesions of maxillary premolars and molars were identified in 16 subjects, 21 sinuses, and 58 teeth. Bone edema and PAI scores were significantly higher using MRI as compared to OPT and AP (*p* < 0.05). Using the STIR sequence, a significant association of PAI score > 1 and the presence of mucosal swelling in the maxillary sinus was detected (*p* = 0.03).

**Conclusion:**

Periapical inflammation and maxillary mucositis could be visualized using STIR imaging. The use of MRI may help detect early, subtle inflammatory changes in the periapical tissues surrounding maxillary dentition. Early detection could guide diagnostic criteria, as well as treatment and prevention.

## Introduction

The association between periapical inflammation of maxillary dentition and odontogenic sinusitis is well documented [1]. In current clinical practice, the diagnosis of odontogenic maxillary sinusitis is based on the detection of apical osteolysis using radiation-based imaging techniques [2, 3]. Periapical osteolysis is a late-stage outcome following chronic inflammation, often from caries-induced pulpitis or endo-perio lesions. Prior to this, lymphocytes and monocytes infiltrate the periapical tissue and initiate an inflammatory cascade of cytokines. These cytokines and mediators disrupt the RANK-RANKL OPG pathway leading to edema [4–8]. The localized edematous change adjacent to the periapical tissues can be visualized using magnetic resonance imaging (MRI) [9].

Current imaging modalities common to clinical practice, such as panoramic radiographs (OPT), periapical radiographs (AP), computed tomography (CT), or cone beam CT (CBCT), allow for visualization of osseous and dental structures with high spatial resolution [10–14]. However, these radiation-based modalities lack the ability to detect intraosseous edema that proceeds bone loss and osteolysis. In contrast, MRI uses water-sensitive imaging sequences that can detect subtle edematous changes. MRI has a higher sensitivity and specificity when compared to radiation-based techniques for detecting periodontal edema and mucositis of the maxillary sinus [15]. In addition, MRI is able to distinguish between mucositis and other infectious etiologies such as empyema, which require different treatments and can lead to severe ascending complications like orbital infection and intracranial abscess formation [16–18]. Recent literature has demonstrated a growing application of MRI for the visualization of dental and osseous structures, all without exposing patients to ionizing radiation [19–24].

There are several fields in dentistry that have utilized MRI. In endodontics, good reproducibility has been shown for visualizing root canals and determining the working length of endodontic files [25, 26]. In periodontics, Probst et al. described water-sensitive STIR sequences to detect bone edema in generalized periodontitis, and Juerchott et al. described the use of MRI for the evaluation of furcation defects [9, 19]. In oral surgery, the use of MRI for implant planning and third molar removal demonstrated good diagnostic accuracy [27–29]. In orthodontics, a recent MRI study showed reliable 3D cephalometric analysis when compared to CBCT [30].

The purpose of this study was to answer the following clinical question: among patients with periapical inflammatory processes in the maxilla, does the use of magnetic resonance imaging (MRI), as compared to conventional periapical (AP) and panoramic radiography (OPT), improve diagnostic accuracy?

## Methods

### Study design

Forty-two subjects who presented to the Department of Periodontology, Ludwig-Maximilians-University Munich, from May to December 2018 with clinical evidence of periodontal disease were included in this study. All subjects presented with a diagnosis of periodontitis. Clinical findings were not available to the MRI examiners, nor were the results of the MRI available to clinical examiners.

The inclusion criteria were the prevalence of mucosal swelling on the MRI, the availability of an existing OPT, and no symptoms of a sinusitis. Exclusion criteria were recent oral surgery procedures, a history of oral maxillofacial syndromes, and standard contraindications for MRI (e.g., implanted pacemaker). Of the 42 subjects who completed the MRI, 16 subjects fulfilled the described inclusion criteria and were enrolled.

The study received an institutional review board approval (Technical University of Munich: Ref.-No.185/18 S and Ludwig-Maximilians-University Munich: Ref.-No. 18–657). The study was retrospectively registered at the DRKS (German Clinical Trials Register, DRKS00020761).

### MRI acquisition

All subjects were scanned with a 3 T MRI scanner (Elition, Philips Healthcare, Best, The Netherlands) at the Department of Diagnostic and Interventional Neuroradiology, Technical University of Munich, using a 16-channel head-neck cervical spine array. Patients were positioned head-first in a supine position. The sequence protocol consisted of a short survey scan for sequence position planning (acquisition time 0:39 min), a three-dimensional (3D) isotropic T2-weighted short tau inversion recovery (STIR) sequence (acquisition time 6:03 min, acquisition voxel size 0.65 × 0.65 × 0.65 mm^3^, repetition time (TR) 2300 ms, echo time (TE) 184 ms, inversion recovery (IR) 250 ms, compressed sense, reduction 5, gap − 0.5 mm, slice oversampling 1.5, water-fat shift (pix)/bandwidth (Hz) 1766/246), and a 3D isotropic T1-weighted fast field echo (FFE) black bone sequence (acquisition time 5:31 min, acquisition voxel size 0.43 × 0.43 × 0.43 mm^3^, TR 10 ms, TE 1.75 ms, compressed sense, reduction 2.3, gap − 0,25 mm, water-fat shift (pix)/bandwidth (Hz) 1503/289).

The 3D T1-weighted black bone sequence was used for the determination of changes within the tooth-supporting alveolar bone associated with periodontitis. The main sequence used for edema detection within the bone was the 3D STIR sequence.

### Analysis of the panoramic radiographs and the periapical radiographs

All OPT and AP imaging was analyzed for the presence of periapical radiolucencies and associated thickening of the maxillary sinus mucosa. For periapical analysis, a periapical index (PAI) score ranging from 1 – healthy, to 5 – severe periapical osteolysis with exacerbating features was used [31] (Table 1).

**Table 1:** Periapical index (PAI) for panoramic and apical radiographs

PAI	Score definition
1	Normal periapical structures
2	Small changes in bone structure
3	Changes in bone structure with mineral loss
4	Apical periodontitis with well-defined radiolucent area
5	Severe apical periodontitis with exacerbating features

### MRI analysis

The detection and measurement of edema were performed in the 3D T2-weighted STIR sequences. The extent of intraosseous edema was measured from cranial to caudal, from medial to lateral, and from ventral to dorsal. The PAI score was graded using a modified version originally implemented for evaluation on CBCT by Estrela et al. [32] (Table 2). Changes of the bone architecture and osteolysis were evaluated on the 3D T1-weighted black bone sequences.

**Table 2:** Modified periapical index (PAI) for magnetic resonance imaging

PAI	Score definition
0	Intact periapical structure
1	Diameter of periapical radiolucency > 0.5–1 mm
2	Diameter of periapical radiolucency > 1.1–2 mm
3	Diameter of periapical radiolucency > 2.1–4 mm
4	Diameter of periapical radiolucency > 4.1–8 mm
5	Diameter of periapical radiolucency > 8 mm

### Assessment of the type and extent of mucosal swelling

The thickness of the mucosa lining the inferior aspect of the maxillary sinus was measured in millimeters using the coronal, axial, and sagittal reformations of the 3D T2-weighted STIR and T1-weighted FFE sequences according to Gürhan et al. and Shanbhag et al. [33, 34]: class 1: 2.1–5 mm; class 2: 5.1–10 mm; and class 3: > 10 mm. Qualitatively, the appearance of the mucosa was classified as “flat” (horizontal thickening of the sinus floor mucosa) or “polypoid” (dome-shaped thickening of the sinus floor mucosa). Image analysis was performed by a radiologist (MD with 4 years radiology experience) and a dentist and radiologist (MD and DMD; 7 years radiology experience, 2 years oral surgery experience). In cases of severe artifacts due to metallic restorations or movement artifacts, single teeth were excluded from further analysis.

### Statistical analysis

SPSS software version 26.0 (SPSS Inc, Chicago, IL, USA) was used for all statistical tests. For continuous variables, the mean and standard error of the mean (SEM) were calculated. Within each experimental group, the normal distribution of data was tested using the Kolmogorov-Smirnov procedure. The Mann-Whitney test was used for independent variables. For categorial data, absolute numbers and the relative frequency within each group are presented. Group comparisons were performed with chi-squared tests with Yates’ correction. Intra- and inter-reader agreements were evaluated. A *p*-value of < 0.05 was considered statistically significant.

## Results

### Patient cohort and clinical findings

Sixteen of the initial 42 subjects were included in this study according to the inclusion and exclusion criteria described in the “Methods” section (mean age 58 years; age range 28–82 years, 10 men and 6 women) with 58 affected teeth.

### Dental findings in panoramic radiographs and dental apical radiographs

Of the 58 teeth, alterations in periapical alveolar bone were detected in 23 teeth using OPT and 24 using AP. No significant association was detected for the extent of apical lesion (neither with a PAI of ≤ 2 nor with a PAI of ≥ 2) or the presence of mucosal swelling (*p* > 0.05). No significant difference was detected on AP films for PAI of 1 compared to PAI of > 1 (*p* > 0.05). For conventional imaging technique, no significant correlation was identified between PAI and mucosal swelling.

### Dental findings on MRI

**Fig. 1:**
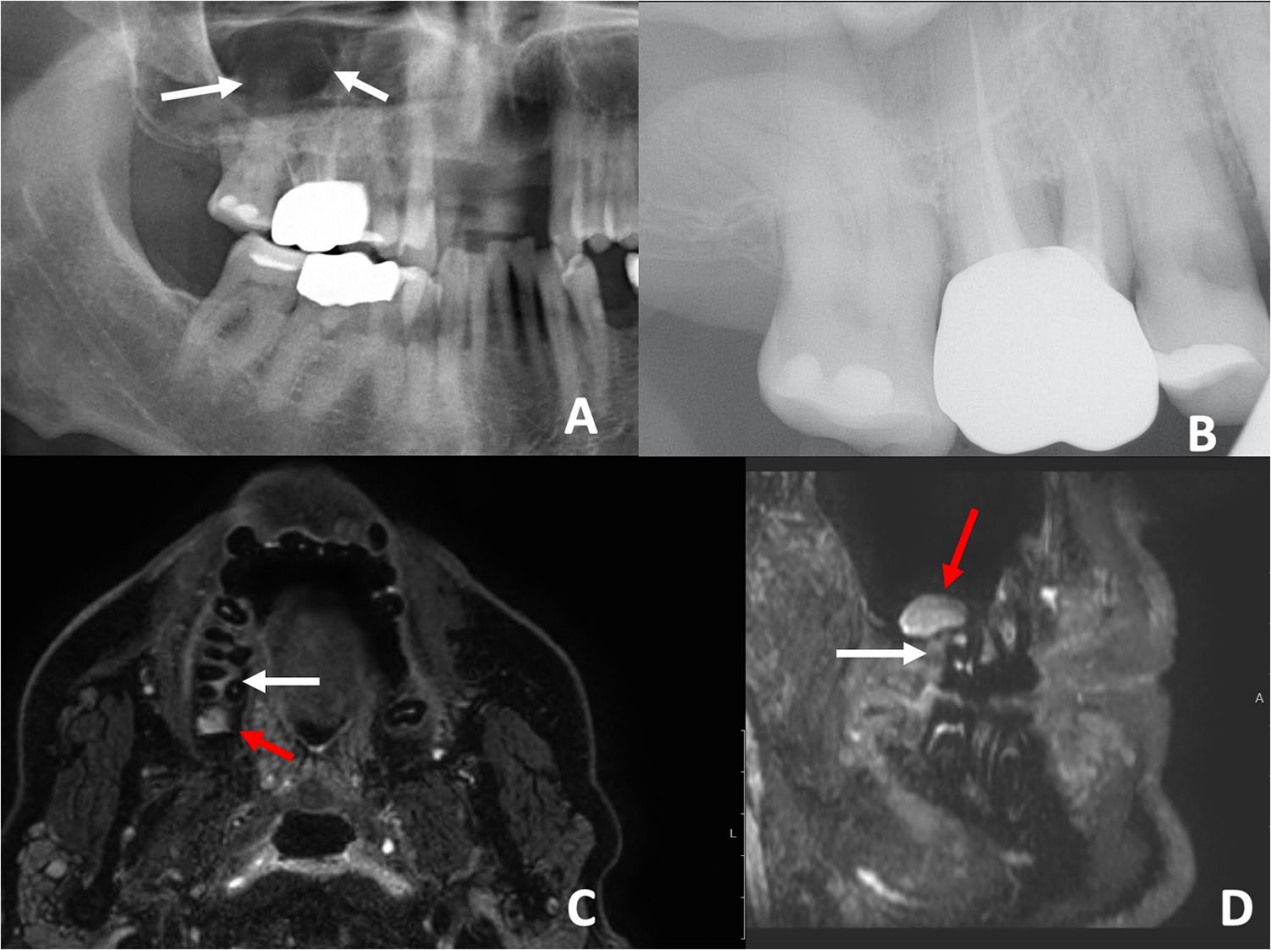
**A** OPT with close spatial relation between the roots of the endodontically treated tooth 16 and the tooth 17. The right maxillary sinus appears to be normal. A circumscribed radiolucency can be seen in the maxillary sinus (white arrows). **B** AP with subtle periodontal space widening around the disto-buccal and the palatinal roots of tooth 17 and the mesio-buccal root of tooth16. **C** In the coronal slice of the STIR sequence, a subtle flat mucosal swelling cranial to the tooth 16 can be detected (red arrow). **D** The sagittal slice shows a subtle STIR-hyperintense bone edema that can be seen distal to the disto-buccal root and in the region of the furcation of tooth 17 (white arrow). The mucosal swelling can be detected at the floor of the maxillary sinus (red arrow)

**Fig. 2:**
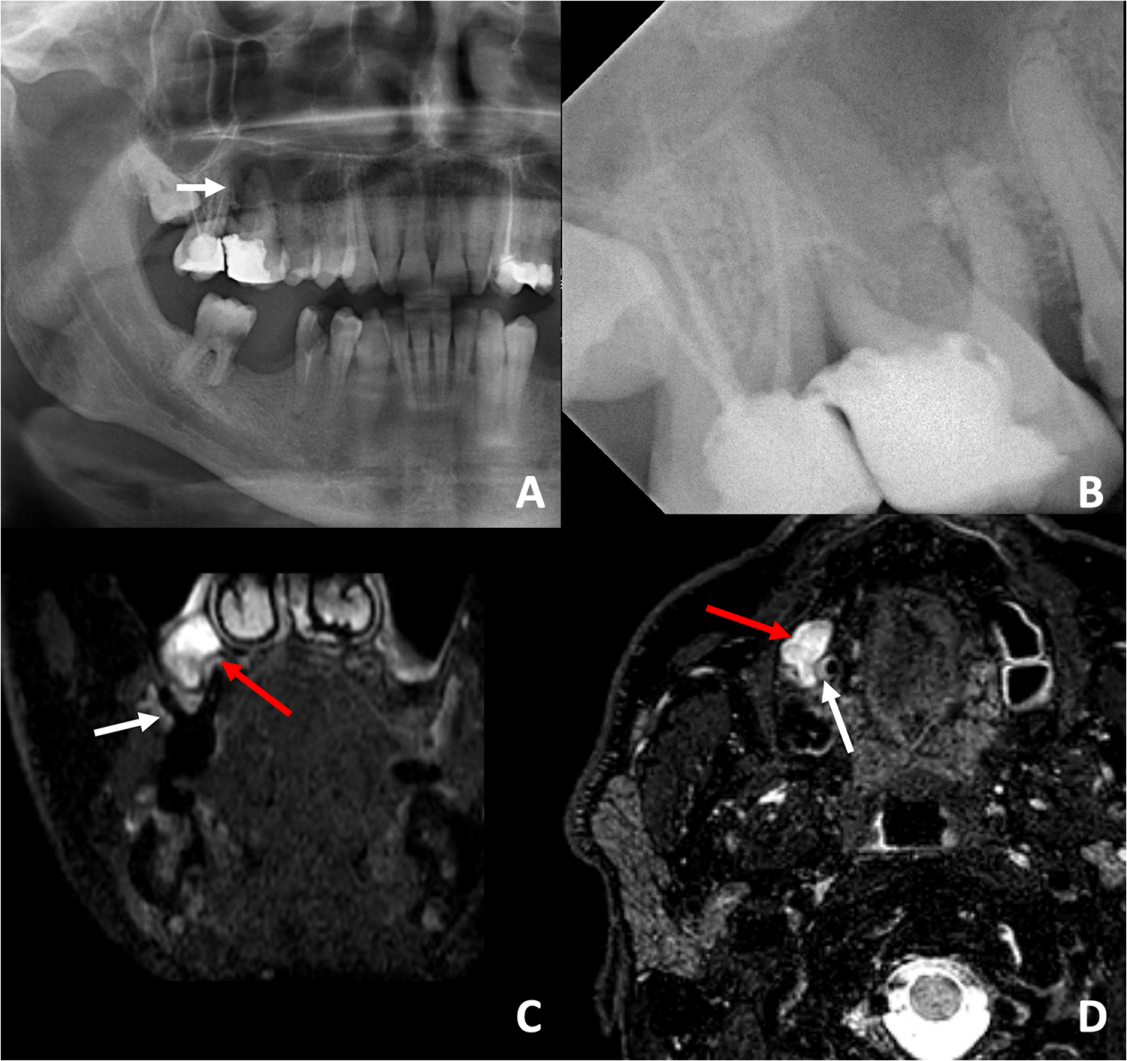
**A** OPT with impression of a periapical radiolucency around the mesio-buccal root of tooth 16 (white arrow). However, artificial air superimposition would also be a differential diagnosis. **B** The AP shows subtle changes around the mesio-buccal and the palatal roots of tooth 16. The endodontically treated tooth 17 also has a widened periodontal ligament space around the mesio-buccal root. Root canal treatment appears sufficient. **C** In the STIR sequence (coronal slice), a large bone edema around the mesio-buccal of tooth 16 can be detected (white arrow), exceeding the corresponding radiolucency displayed in the AP. Additionally, a marked STIR-hyperintense, periapical granuloma is depicted and lifts the Schneiderian membrane more cranial (STIR-hypointense, linear structure, white arrow). Above the Schneiderian membrane, a mucosal swelling can be detected (red arrow). **D** In the axial slice, the full extent of the STIR-hyperintense bone edema (white arrow) can be delineated. The bottom part of the granuloma can be seen (red arrow)

Using the STIR sequence, a significant association of a PAI score > 1 and the presence of mucosal swelling in the maxillary sinus was detected (*p* = 0.03). However, the extent and type of mucosal swelling revealed no statistically significant association with the PAI score detected on MRI. For example, Fig. 1 displays subtle periapical edema at the buccal roots of a second molar associated with flat mucosal thickening. Figure 2 displays an extensive apical granuloma that can be seen on the STIR sequence with associated subtle bone edema. Descriptive findings are given in Table 3.

**Table 3:** PAI and edema measurements on MRI and OPT

Overall				
	OPT	Dental radiograph	3D STIR	*p*-value
Mean extent of radiolucency and bone edema (mm)	1.3 ± 1.6	0.8 ± 1.8	2.4 ± 1.5	< 0.05
Mean PAI scores	2.1 ± 0.7	1.8 ± 0.5	2.1 ± 0.6	< 0.05

Data are presented as means ± standard deviations

PAI score OPT and dental radiograph (1 = healthy, 5 = severe periapical osteolysis), PAI score MRI (0 = healthy, 5 = diameter of periapical radiolucency > 8 mm)

### Mucosal pathologies detected on MRI

In 16 subjects, 21 maxillary sinuses demonstrated mucosal swelling. The predominant type of mucosal swelling was the flat type (18 of 21 maxillary sinuses), whereas only 3 subjects showed a polypoid configuration. In eight subjects, the bottom thickness of the maxillary mucosa could be classified as class 1, in 4 subjects the thickness corresponding to class 2, and in 9 subjects the thickness corresponded to more than 10 mm (class 3).

In OPT as well as in AP, only two subjects with class 1 thickness were detected. Half of the cases assigned to class 2 were detected on both conventional radiation-based imaging techniques.

### Intra- and inter-reader agreement

The inter-reader agreement for the PAI scores on the OPT, dental radiographs, and MRI was substantial to almost perfect (OPT: *κ* 0.94, 95% confidence interval 0.93–1.00; dental radiograph: *κ* 0.96, 95% confidence interval 0.92–1.00; 3D STIR *κ* 0.95, 95% confidence interval 0.94–1.00). The inter-reader agreement for the detection of mucosal swelling was also substantial to almost perfect (*κ* 0.93, 95% confidence interval 0.90–1.00). For intra-reader reliability, both readers reassessed the images of 10 patients after at least 8 weeks, showing very good agreement between the two time points (*κ* 0.97, 95% confidence interval 0.94–1.00).

## Discussion

In this study, we showed that periapical edema of maxillary molars and premolars detected on MRI was associated with the presence of mucosal thickening of the maxillary sinus. Notably, pathological periapical findings on MRI were not significantly associated with the severity of inflammatory reaction. However, subtle changes of PAI could be detected by MRI, prior to the onset of osteolysis that would be detectable on conventional radiography. Thus, MRI seems to be suitable for the early detection of periapical changes with associated mucosal swelling that are not present on OPT or AP.

This project sought to compare MRI, which is considered to be an advanced imaging modality, to standard-of-care imaging that is routinely available in a dental office. In more advanced surgical or hospital-based settings, the use of CT and CBCT are also available to analyze more precise anatomical structures in demanding clinical situations [10–12, 35]. The downside of all X-ray-based imaging techniques is the individual’s exposure to ionizing radiation harboring the risk of stochastic radiation effects with associated damage of the deoxyribonucleic acid (DNA). That being said, every clinician should follow the ALARA principle (“as low as reasonably achievable”) when making the choice for an imaging technique [24].

In contrast to radiation-based imaging techniques, MRI is based on non-ionizing radiation using the different magnetic properties of hydrogen nuclei contained in water and fat for generating images. This is the reason why MRI is able to depict soft tissues with a much higher contrast than conventional cross-sectional imaging modalities. Recently, Van der Cruyssen et al. and Juerchott et al. have shown that by optimizing sequence protocols, direct visualization of small trigeminal branches and even complex structures like the dental pulp is possible, which improves our understanding of nerve physiology in vivo [36–39]. Furthermore, using T1-based imaging or ultrashort or zero echo time sequences, even the visualization of hard tissues like the mandibular bone and pathological alterations in the course of periodontitis or osteonecrosis has been feasible [9, 27, 40, 41].

STIR imaging has been proven to detect bone edema as a marker for inflammatory changes in hard and soft tissue [42, 43]. Hyperintense signal alterations using STIR sequences can detect changes to the osseous matrix even before T1-weighted sequences. By applying STIR sequences to dental medicine, apical inflammation that was previously undetectable on X-ray imaging and clinically silent can now be detected. The early identification of these inflammatory processes could allow intervention prior to exacerbations and chronic disorders. Furthermore, follow-up imaging with close intervals is possible to validate the suspected diagnosis and monitor progression.

In a recent study, STIR sequences were utilized to detect bone edema and showed a good correlation to clinical parameters in periodontitis [9]. The authors previously attempted to raise clinician awareness to MRI capabilities in detecting early osseous and inflammatory changes related to odontogenic infections. A survey among otolaryngology residents conducted in the USA showed that there is a lack of knowledge in regard to odontogenic sinusitis, which can have serious risks ranging from orbital infections to intracranial spread [2, 16, 18]. In this context, applying MRI in a clinical setting using dedicated water-sensitive sequences could help identify teeth at risk or with already detectable edema before inflammatory spread to other anatomic structures. Thus, MRI can depict soft tissue and intraosseous inflammatory changes in early stages, which gives clinicians the opportunity to review treatment options [9, 44]. In cases of associated tooth decay, timely initiation of excavation with subsequent partial pulpotomy or pulpectomy could prevent bacterial spread to the alveolar bone and shorten intervals to definitive root canal filling. However, there are certain settings that hamper the specificity of detected hyperintensities derived from STIR sequences. Patients with parafunctions and bruxism not only exhibit dental attrition, but also expose the periodontium and the adjacent alveolar bone to pathological loading, which might be mirrored by periapical signal alterations. For interpreting the obtained images of MRI, advanced knowledge of anatomy and sequence peculiarities is essential, which requires a good communication of the clinician and the radiologist to maximize diagnostic accuracy and improve patient treatment.

This study supports the use of MRI as a complementary imaging modality in the clinical evaluation of periapical inflammatory changes. It has to be stated that for optimal diagnostic quality, the combination of MRI and conventional radiography is mandatory depending on dental examination and derived indication. For direct visualization of osseous and dental structures and pathologies OPT, AP and bitewing imaging is and will be essential in dental diagnostics.

The conducted study has several limitations. As a feasibility study limited to a small cohort, the *n* value was small and did not allow for advanced statistical analysis. The presented results are limited and need to be validated in larger cohorts. Second, in subjects that have subtle changes of the mucosal thickness, artifacts arising from the air-filled maxillary sinus can reduce image quality. Third, although the applied sequence protocol was rather short, image quality could be hampered by motion (e.g., chewing or mouth breathing).

## Conclusion

Periapical inflammation and maxillary mucositis could be visualized using STIR imaging. The use of MRI may help detect early, subtle inflammatory changes in the periapical tissues surrounding maxillary dentition. Early detection could guide diagnostic criteria, as well as treatment and prevention.

## Data Availability

All source data are stored at the Department of Neuroradiology, Technical University of Munich, Munich, Germany. We invite parties interested in collaboration and data exchange to contact the corresponding author directly.
